# Suppression of Her2/Neu mammary tumor development in *mda-7/IL-24* transgenic mice

**DOI:** 10.18632/oncotarget.6046

**Published:** 2015-10-09

**Authors:** You-Jun Li, Guodong Liu, Lei Xia, Xiao Xiao, Jeff C. Liu, Mitchell E. Menezes, Swadesh K. Das, Luni Emdad, Devanand Sarkar, Paul B. Fisher, Michael C. Archer, Eldad Zacksenhaus, Yaacov Ben-David

**Affiliations:** ^1^ Department of Anatomy, Norman Bethune College of Medicine, Jilin University, Changchun, Jilin, China; ^2^ Department of Nutritional Sciences, University of Toronto, Toronto, Ontario, Canada; ^3^ Department of Medical Biophysics, University of Toronto, Toronto, Ontario, Canada; ^4^ Division of Biology, The Key Laboratory of Chemistry for Natural Products of Guizhou Province and Chinese Academy of Sciences, Guiyang, China; ^5^ Toronto General Research Institute - University Health Network, Toronto, Ontario, Canada; ^6^ Department of Human and Molecular Genetics, VCU Institute of Molecular Medicine, VCU Massey Cancer Center, Virginia Commonwealth University, School of Medicine, Richmond, Virginia, USA

**Keywords:** mda-7/IL-24, HER2, breast cancer, prevention, mouse model

## Abstract

Melanoma differentiation associated gene-7/interleukin-24 (*mda-7/IL-24*) encodes a tumor suppressor gene implicated in the growth of various tumor types including breast cancer. We previously demonstrated that recombinant adenovirus-mediated *mda-7/IL-24* expression in the mammary glands of carcinogen-treated (methylnitrosourea, MNU) rats suppressed mammary tumor development. Since most MNU-induced tumors in rats contain activating mutations in *Ha-ras,* which arenot frequently detected in humans, we presently examined the effect of MDA-7/IL-24 on Her2/Neu*-*induced mammary tumors, in which the RAS pathway is induced. We generated tet-inducible MDA-7/IL-24 transgenic mice and crossed them with Her2/Neu transgenic mice. Triple compound transgenic mice treated with doxycycline exhibited a strong inhibition of tumor development, demonstrating tumor suppressor activity by MDA-7/IL-24 in immune-competent mice. MDA-7/IL-24 induction also inhibited growth of tumors generated following injection of Her2/Neu tumor cells isolated from triple compound transgenic mice that had not been treated with doxycycline, into the mammary fat pads of isogenic FVB mice. Despite initial growth suppression, tumors in triple compound transgenic mice lost *mda-7/IL-24* expression and grew, albeit after longer latency, indicating that continuous presence of this cytokine within tumor microenvironment is crucial to sustain tumor inhibitory activity. Mechanistically, MDA-7/IL-24 exerted its tumor suppression effect on HER2^+^ breast cancer cells, at least in part, through PERP, a member of PMP-22 family with growth arrest and apoptosis-inducing capacity. Overall, our results establish *mda-7/IL-24* as a suppressor of mammary tumor development and provide a rationale for using this cytokine in the prevention/treatment of human breast cancer.

## INTRODUCTION

Breast cancer is a devastating disease, constituting more than 25% of the total number of new cancer cases diagnosed annually among women throughout the world [1]. Amplification or over-expression of the receptor tyrosine kinase HER2 induces one of the most aggressive forms of breast cancer [2]. Although anti-HER2 therapy provides some improvement in disease free survival, drug-refractory metastatic disease is almost invariably fatal [3]. The development of new treatments or prophylactic means to reduce the incidence of this disease is, therefore, of great interest.

Melanoma differentiation associated gene-7 was originally identified and cloned using subtraction hybridization from metastatic human melanoma cells that were induced to undergo terminal differentiation and lose proliferative and tumorigenic properties [4]. *mda-7/IL-24* is a member of the IL-10 family of cytokines, and named IL-24 (*mda-7/IL-24*) [5-8]. It encodes a secreted tumor suppressor protein of 206 amino acids, which inhibits the growth of diverse human cancers, without harming normal cells or tissues [4, 9-11]. *mda-7/IL-24* displays restricted expression in normal and cancer cells, observed in melanocytes and subsets of T-cells, but not in the majority of normal or cancer cells [8, 11, 12]. Expression of this cytokine can be induced in both immune and non-immune cells through interaction of the secreted protein with IL-20R1/IL-20R2 and IL-22R1/IL-20R2 cell surface receptors inducing autocrine and paracrine secretion of this cytokine and cancer-specific apoptosis [8, 13-15]. *mda-7/IL-24* displays multiple properties that support its ability to serve as a cancer suppressor gene, including an ability to selectively induce apoptosis and toxic authophagy in a broad-spectrum of cancer cells, potent “bystander” antitumor activity, anti-angiogenic activity, immune modulatory properties and synergy with conventional therapeutics (radiation, chemotherapy and antibody-based therapies) [rev. 7, 8, 15, 16]. *mda-7/IL-24* has shown significant anti-tumor activity *in vivo* in pre-clinical animal models including multiple human tumor xenografts in nude mice [17-24] and recently in prostate cancer genetically engineered mouse (GEM) models [25, 26]. When *mda-7/IL-24* was administered to patients with advanced cancers by repeat intratumoral injection using a replication incompetent adenovirus (Ad.*mda-*7; INGN 241) it was shown to be safe and induced a 44% response rate, promoting cancer apoptosis in injected lesions [27-32]. A potential link between *mda-7/IL-24* and specific autoimmune diseases such as psoriasis and rheumatoid arthritis has been suggested [33].

The mechanism by which *mda-7*/IL-24 exerts its tumor suppressor activity has been extensively studied in the past decade [rev. in 7, 8, 15, 16, 28]. Several studies indicate that *mda-7/IL-24* selectively induces apoptosis in cancer cells without harming normal cells, by promoting an endoplasmic reticulum (ER) stress response [34-37]. Recent studies also demonstrate selective apoptosis by Suppressor of AP-1, induced by an IFN (SARI)-dependent mechanism [14]. Although the majority of studies have emphasized solid tumors, *mda-7/IL-24* also induces ER stress and mitochondrial apoptosis pathway in human acute myeloid leukemia (AML) and chronic lymphocytic leukemia (CLL) [38, 39]. A potential role for ceramide production (ceramide synthase, PP2A) and generation of reactive oxygen species as a consequence of induction of ER stress by *mda-7/IL-24* in tumor cells has also been demonstrated [7, 8, 40-42]. As emphasized in numerous reviews, *mda-7/IL-24* can elicit cancer-selective killing through multiple pathways, including those involving modification of signaling pathways and molecules (including BiP/GRP78, GRP94, P-PKR, PERK, P-p38 MAPK, CD95, Bax, Bak) that can lead to activation of caspase 9/3 resulting in mitochondrial-mediated apoptosis, through death-receptor mediated killing or toxic authophagy [rev. in 7, 8, 15, 16, 28]. Despite this progress, there is only limited direct evidence for tumor suppressor activity by MDA-7/IL-24 in immune-competent transgenic mice [25, 26].

We have previously shown that growth suppression by *mda-7/IL-24* is associated with transcriptional up-regulation of p27^Kip1^ via Stat3 activation in breast cancer cells [43]. Furthermore, we showed that β4 integrin is a downstream target of *mda-7/IL-24* [43]. More recently, we identified the growth arrest-specific gene 3 (*gas3*) as a downstream target of *mda-7/IL-24* [44]. We further demonstrated that the induction of *gas3* by *mda-7*/IL-24 inhibits attachment and proliferation of tumor cells *in vitro* and *in vivo* by blocking interaction of β1 integrin with fibronectin [44].

We, and others, have shown that delivery of *mda-7*/*IL-24* via an adenovirus vector can efficiently inhibit growth of diverse cancer cells *in vivo* and *in vitro* [rev. in 7, 8], including breast cancer [10, 17, 43-49]. Specifically, we demonstrated significant inhibition of tumor development following injection of an adenovirus carrying *mda-7/IL-24* into the main mammary ducts of rats induced to develop breast cancer by treatment with methylnitrosourea (MNU) [44]. Most MNU-induced tumors in rats contain activating mutations in the *Ha-ras* oncogene [50]. Since *Ha-ras* mutations are not frequently detected in humans, in the present study we investigated whether *mda-7/IL-24* could inhibit development of Her2/Neu-induced breast cancer. We generated tet-inducible *mda-7/IL-24* transgenic mice to investigate whether *mda-7/IL-24* expression could suppress development of Her2/Neu mammary tumors in compound immunocompetent transgenic mice. Our results provide a rationale for the prophylactic use of *mda-7/IL-24* in the prevention as well as applications for therapy of HER2^+^ breast cancer.

## RESULTS

### Generation of doxycycline-inducible *mda-7/IL-24* transgenic mice

**Figure 1:**
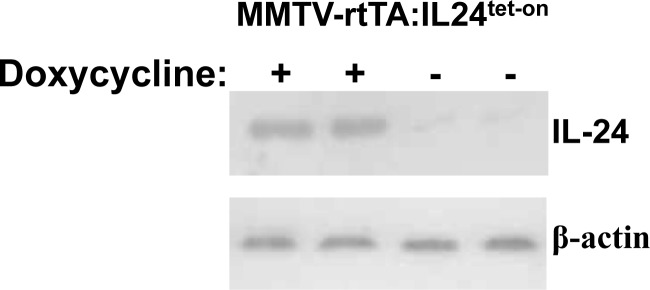
**A.** Schematic of generation of ^IL24tet-on^ transgenic mice and genetic cross to create tet-inducible MMTV-rtTA:IL24^tet-on^ mice. **B**. Western blot of MDA-7/IL-24 expression in mammary glands isolated from two MMTV-rtTA:IL24^tet-on^ double transgenic mice fed regular or doxycycline-containing chow.

To generate transgenic mice that can be induced to over-express MDA-7/IL-24 specifically in the mammary gland, we placed the *mda-7/IL-24* gene under control of a tet-inducible Ptet^OS^-4264 promoter [51]. The plasmid was injected into pronuclei of FVB mouse oocytes, and transgenic mice, referred to as IL24^tet-on^, were identified by PCR. To induce expression of *mda-7/IL-24* specifically in mammary epithelium, IL24^tet-on^ mice were mated with MMTV-rtTA mice (Figure 1A). The latter mice express the reverse tetracycline-dependent transactivator rtTA under control of the mouse mammary tumor long terminal repeat (MMTV-LTR) [52]. Transgene expression in this system can be rapidly induced by feeding mice doxycycline-containing chow (625 mg/kg), is highly mammary specific, and is essentially undetectable in the un-induced state [52].

To test for *mda-7/IL-24* inducibility, we fed MMTV-rtTA:IL24^tet-on^ double compound transgenic females regular or doxycycline-containing chow. Mammary glands were harvested and subjected to western blot analysis (Figure 1B). In contrast to control mice that expressed very low levels of *mda-7/IL-24*, doxycycline induced robust *mda-7/IL-24* expression (53-fold increase over background).

### Transgenic *mda-7/IL-24* expression suppresses Her2/Neu tumor formation

**Figure 2:**
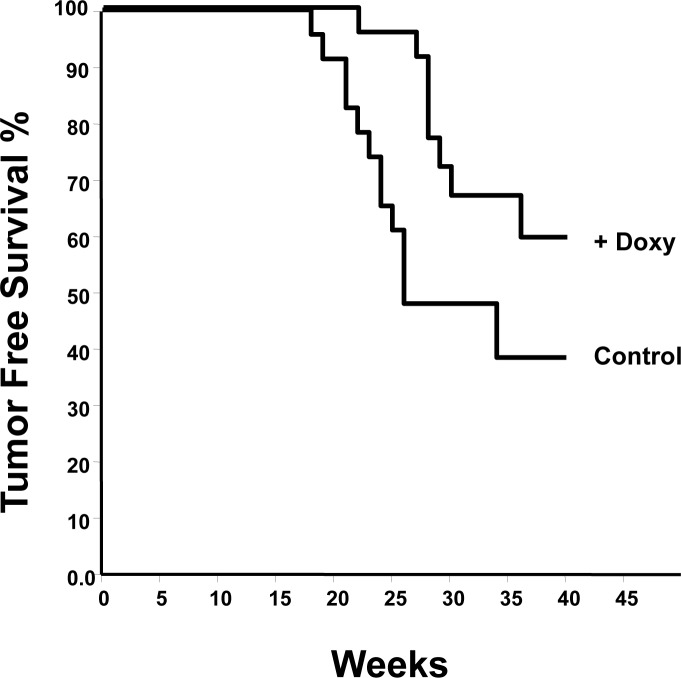
Kaplan-Meier analysis of tumor development in MMTV-rtTA:IL24^tet-on^:MMTV-Her2/neu triple compound transgenic mice fed regular chow (controls) or chow containing doxycycline Hazard ratio 2.1 (*P* = 0.005).

To determine the effect of *mda-7/IL-24* on tumor development, we next crossed MMTV-rtTA:IL24^tet-on^ mice with MMTV-Her2/neu transgenic mice [53]. A group of 23 MMTV-rtTA:IL24^tet-on^:MMTV-Her2/neu triple compound transgenics was fed chow-containing doxycycline, while a similar size group was fed regular chow. Mice were monitored weekly for signs of tumor formation. Kaplan-Meier tumor-free survival curves for the two groups were generated (Figure 2). It is clear that induction of *mda-7/IL-24* significantly reduced tumor progression (*P* = 0.005) with hazard ratio of 2.7.

**Figure 3:**
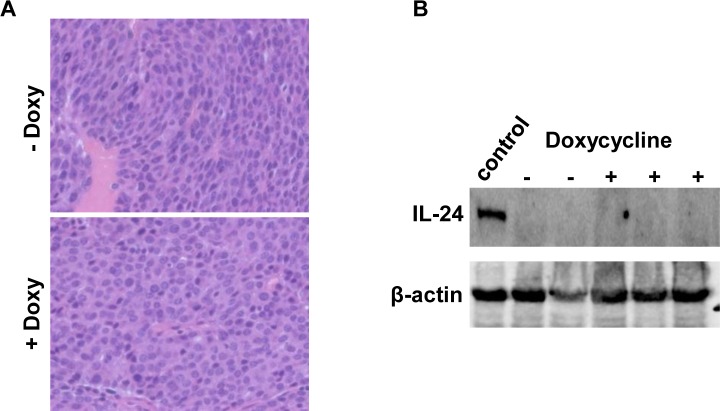
**A.** H&E staining of tumors from MMTV-rtTA:IL24^tet-on^:MMTV-Her2/neu triple transgenic mice fed regular chow or chow containing doxycycline. Original magnification, 400X. **B.** Western blot demonstrating lack of expression of MDA-7/IL-24 in tumors that arose in either doxycycline-treated or untreated mice. Each lane contained 50μg protein; the positive control lane contained protein isolated from the mammary glands of double transgenic mice fed doxycyline as shown in Figure 1A.

Tumors from both groups were fixed for histological analysis. Those from control mice not administered doxycycline were typical, poorly differentiated adenocarcinomas as previously characterized in these animals (Figure 3A). Tumors from doxycycline-treated mice were indistinguishable from the control group. Western analysis showed no MDA-7/IL-24 expression in tumors from either doxycycline-treated mice or untreated mice (Figure 3B). This result was confirmed in Western blots performed with a three-fold increase (150 μg) in the amount of protein loaded per lane (data not shown).

### Effect of mda-7/IL-24 expression on Her2/Neu tumor growth

We next asked whether induction of *mda-7/IL-24* would reduce growth of pre-existing tumors. To this end, Her2/Neu tumors were allowed to form in triple compound transgenic mice fed normal chow. Once tumors were detected, half the mice were shifted to a doxycycline-containing diet. We found that tumors in these mice continued to develop over a 6-week period at the same rate as those in control mice (data not shown). These results are consistent with the observation above (Figure 3B) that once tumors are formed in triple compound transgenic mice, they shut down *mda-7/IL-24* expression and no longer respond to doxycycline.

**Figure 4:**
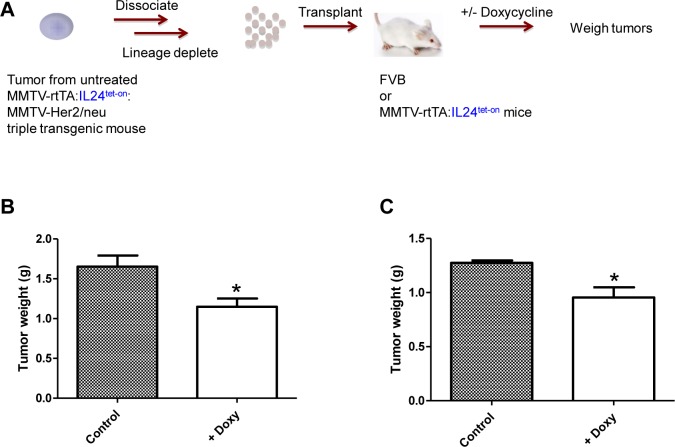
**A.** Schematic of experimental design. **B.** Effect of *mda-7/IL-24* expression on the growth of tumor cells from MMTV-rtTA:IL24^tet-on^:MMTV-Her2/neu triple transgenic mice fed regular chow, which were dissociated into single cells and transplanted into FVB mice. The FVB mice were then fed regular chow or chow containing doxycycline for 12 weeks. Tumors were dissected and weighed (*n* = 8, *P* = 0.0275; unpaired Student t-test). **C.** Effect of MDA-7/IL-24 expression on growth of tumor cells from MMTV-rtTA:IL24^tet-on^:MMTV-Her2/neu triple transgenic mice fed regular chow, which were dissociated into single cells and transplanted into MMTV-rtTA:IL24^tet-on^ double transgenic mice. The double transgenic mice were then fed regular chow or chow containing doxycycline for 12 weeks before MMTV-rtTA:IL24^tet-on^:MMTV-Her2/neu tumors were dissected and weighed (*n* = 8, *P* = 0.0152; unpaired Student *t*-test).

Next we determined whether MDA-7/IL-24 expression could suppress growth of pre-existing tumor cells *in vivo*. To this end, lineage-depleted tumor cells were isolated from MMTV-rtTA:IL24^tet-on^:MMTV-Her2/neu triple compound transgenic mice that had not been treated with doxycycline, then injected into the mammary fat pads of FVB mice (Figure 4A). Treatment of recipient mice with doxycycline to induce *mda-7/IL-24* expression significantly inhibited tumor growth compared with untreated mice (*P* < 0.03) (Figure 4B). We obtained a similar result when the recipient mice were MMTV-rtTA:IL24^tet-on^ double compound transgenics (*P* < 0.02) (Figure 4C).

### *mda-7/IL-24* regulates p53 apoptosis effector related to PMP-22 (PERP) in breast cancer cells

**Figure 5:**
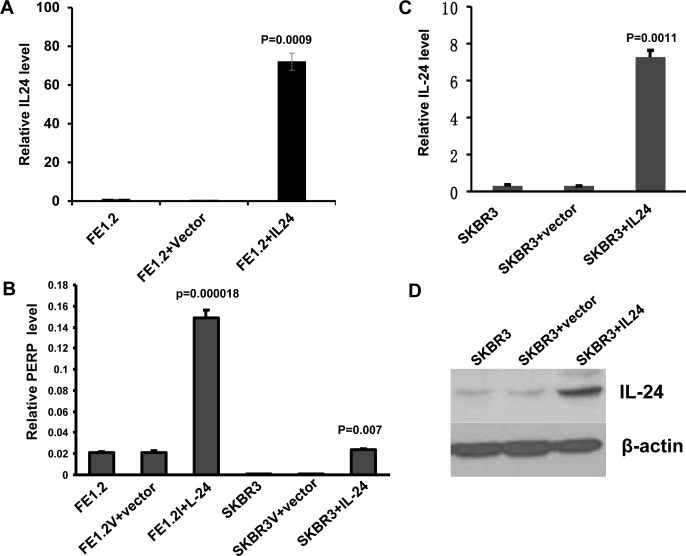
MDA-7/IL-24 directly induces PERP transcription **A.** Expression of mda-7/IL-24 by Q-rtPCR in FE1.3 cells alone and in FE1.2 cells transfected with vector (FE1.2+vector) or MSCV-IL-24 (FE1.2+IL-24), as previously described (44). **B.** Expression of PERP by Q-rtPCR in FE1.2 or SKBR3 cells transfected with vector or *mda-7/IL-24* retroviruses. **C.** Expression of MDA-7/IL-24 by Q-rtPCR in SKBR3 cells transfected with vector or *mda-7/IL-24* retroviruses. **D.** Expression of MDA-7/IL-24 by Western blotting in SKBR3 cells transfected with empty vector or *mda-7/IL-24* retroviruses.

In a recent study, we identified *gas3/pmp-22* as a downstream target of *mda-7/IL-24* that mediates some of the growth inhibitory effects of this cytokine [44]. In the microarray analysis used to identify GAS3 [44], we also observed increased expression of another member of the GAS-3/PMP-22 family - p53 apoptosis effector related to PMP-22, PERP [54]. Here we show that the level of *perp* transcripts as determined by Q-rt-PCR was significantly higher in FE1.3 cells, expressing high levels of endogenous *mda-7/IL-24*, or in FE1.2 cells over-expressing *mda-7/IL-24* (FE1.2+IL-24), than in control FE1.2+vector cells (Figure 5A and 5B). FE1.2 and FE1.3 are both breast cancer cell lines previously isolated from MNU-treated rats [55]. Over-expression of *mda-7/IL-24* in the HER2^+^ breast cancer cell line SKBR3 (Figure 5C and 5D) also induced *perp* mRNA levels (Figure 5B).

**Figure 6:**
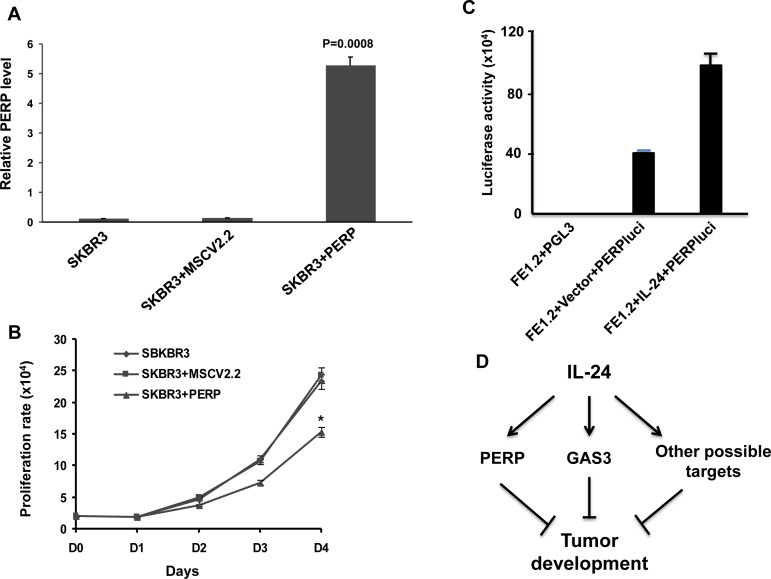
MDA-7/IL-24 regulates PERP expression **A.** Expression of PERP by Q-rtPCR in SKBR3 cells transfected with vector or MSCV-perp retroviruses. **B.** Growth rate of SKBR3, SKBR3 cells transfected with vector (SKBR3-vector) or perp cDNA (SKBR3-prep). * denotes *P* = 0.0074 at day 4 comparing SKBR3-vector with SKBR3-prep. **C.** Luciferase activity in FE1.2, FE1.2+vector and FE1.2+MSCV-IL-24 after transfection with a luciferase vector driven by the PERP promoter (PERPluci) as previously described (75). **D.** A model for *mda-7/IL-24* suggesting that it induces PERP (this study), GAS3 (44) and likely other factors that collectively suppress tumor development.

To determine the effect of over-expressing PREP on growth of SKBR3 cells, we used a retroviral expression vector (Figure 6A). *Prep* overexpression significantly inhibited proliferation compared to untransfected or vector alone (Figure 6B). To further investigate *perp* regulation by MDA-7/IL-24, a luciferase vector driven by the *perp* promoter (PERPluci) was transiently transfected into FE1.2+IL-24 and FE1.2+vector cells. This led to a significant up-regulation of luciferase activity in PERPluci-transfected cells in response to MDA-7/IL-24 (Figure 6C). These results demonstrate that members of the PMP-22 family of tumor suppressor genes play an important role in *mda-7/IL-24*-mediated growth inhibition of HER2^+^ breast cancer cells (Figure 6D).

## DISCUSSION

We report that mammary tumor development was significantly inhibited in MMTV-rtTA:IL24^tet-on^:MMTV-Her2/neu triple compound transgenic mice in which *mda-7/IL-24* was over-expressed by means of a doxycycline inducible promoter. Moreover, transplantation of tumors induced in the triple compound transgenic mice that were not treated with doxycycline into FVB or MMTV-rtTA:IL24^tet-on^ double compound transgenic mice resulted in tumors that grew significantly slower after treatment with the *mda-7/IL-24* inducer. These results provide direct evidence for tumor inhibitory activity of MDA-7/IL-24 in Her2/Neu-induced breast cancer.

While our results demonstrated a robust tumor inhibition in triple compound transgenic mice after transgene induction, the expression of *mda-7/IL-24* was found to be negligible in tumors harvested prior to euthanasia. This observation suggests that *mda-7/IL-24* expression subsides during tumor development. This conclusion is supported by our findings that tumor growth was not inhibited in triple compound transgenic mice that had not been treated with doxycycline but were then treated with doxycycline for 6 weeks (not shown). Thus, *mda-7/IL-24* expression appears to be shut down during tumor evolution, explaining the delayed, rather than complete, suppression of tumor formation in the doxycycline-treated mice, and the robust growth of these tumors once formed. Since *mda-7/IL-24* acts as tumor suppressor, loss of transgene expression, perhaps as a result of limited delivery of doxycycline to the tumor mass or epigenetic silencing, may promote selection of faster growing cancer cells that eventually dominate. This result raises the possibility that future cancer therapy may require a delivery mechanism that ensures persistent expression/delivery of this cytokine to the tumor microenvironment. Alternatively, combining *mda-7/IL-24* with a second therapeutic agent that may directly target the tumor, activate the immune system and/or affect the tumor microenvironment might promote a more sustained therapeutic response [7, 15-17, 24-26, 48, 56]. A recent study suggests that phosphorylation of MDA-7/IL-24 is required for its anti-cancer activity in a single lung cancer cell line, H1299 [57]. The authors suggest that *phosphomimetic MDA-7/*IL-24 or pharmacological induction of phosphorylation of endogenous or exogenous MDA-7/IL-24 may further promote its anti-tumor effects. An alternate strategy could employ high throughput screening and combinatorial chemistry approaches, to identify small molecules that can either stabilize *mda-7/IL-24* mRNA, enhance translation of *mda-7/IL-24* mRNA into protein or promote IL-20R1/IL-20R2 or IL-22R1/IL-20R2 receptor activation to induce endogenous *mda-7/IL-24* mRNA production and protein in target cells [58-68].

Transplantation of lineage-depleted tumor cells from triple compound transgenic mice fed regular chow into FVB or MMTV-rtTA:IL24^tet-on^ double compound transgenic mice treated with doxycycline inhibited subsequent tumor growth. These results are consistent with the notion that high local MDA-7/IL-24 is growth suppressive, but that once tumor cells grow, MDA-7/IL-24 expression in specific contexts may be shut down through mechanisms that are currently not fully understood.

In the past decade, various downstream target genes were suggested as potential mediators of growth suppression by *mda-7/IL-24* [rev. in 7, 8, 15, 16]. We recently discovered the GAS-3/PMP-22 tumor suppressor gene as a novel target for this cytokine [44]. Our identification here of the MDA-7/IL-24 target PERP, another member of the GAS-3/PMP-22 family with strong tumor inhibitory activity, further highlights the importance of this gene family in malignant transformation. Since PERP is also a direct target of p53, our data suggest that MDA-7/IL-24 can induce growth arrest independently of p53, as supported by previous work [69-71], and may, therefore, be a useful inhibitor of tumors harboring TP53 mutations or deletions.

The anti-tumor effects of *mda-7/IL-24* are likely mediated not only by autonomous cellular mechanisms through its downstream targets (Figure 6, model), but also through induction of an immune response. This is exemplified in the transplantation experiment shown in Figure 4 in which IL-24-transgenic tumor cells were injected into immuno-competent FvB mice, but an immune response may also play a role in the transgenic mouse experiment (Figure 3). In an accompanying paper by Menezes et al. [72] three distinct transgenic mouse models of breast cancer were utilized to determine the role of MDA-7/IL-24 in mammary tumorigenesis. The findings in that paper provide further proof of the relevance of MDA-7/IL-24 in tumor suppression in mouse models with an intact immune system. The findings further demonstrate the role of MDA-7/IL-24 in eliciting an antitumor immune response and show that adenovirus mediated *mda-7/IL-24* delivery induces infiltrating CD8^+^ T cells expressing high levels of levels of IFN-γ expression [72]. Thus, induction of exogenous *mda-7/IL-24* may not only suppress cell proliferation directly but may also elicit an immune response in immune-competent animals, further attenuating tumor growth.

In summary, our data provides strong support for the role of *mda-7/IL-24* as a suppressor of Her2/Neu-induced breast cancer and should encourage development of a therapy based on continuous *mda-7/IL-24* delivery into tumor cells, and based on a preponderance of data in the literature in combination with other therapeutic agents. Although direct injection of recombinant IL-24 or viral-based deliveries are possible, an appealing direction is to identify novel drugs that can upregulate or stabilize endogenous levels of this cytokine tumor suppressor.

## MATERIALS AND METHODS

### Generation of Tg mice

*mda-7/IL-24* mRNA, isolated from our rat mammary tumor cell line FE1.3 [55] was used to derive cDNA that was cloned into the EcoRI site of plasmid Ptet^OS^-4264 [51] kindly supplied by Dr. D. Dumont, Sunnybrook Health Sciences Centre, Toronto, Canada. This construct was microinjected into pronuclei of FVB mouse oocytes, which were then implanted into pseudopregnant recipients by the Toronto Centre for Phenogenomics (www.phenogenomics.ca). Pups were screened for the presence of *mda-7/IL-24* at 2-3 weeks of age using the forward primer ATGGTTCTCCAAGCCTTCCT and reverse primer CCCTGAGGACAACAGGGATA. In order to express *mda-7/IL-24* specifically in the mammary gland, transgenic founders were crossed with MMTV-rtTA mice in an FVB background [52] kindly supplied to us by Dr. L. A. Chodosh, University of Pennsylvania School of Medicine. The double compound transgenics were identified using the forward primer ATCCGCACCCTTGATGACTCCG and the reverse primer GGCTATCAACCAACACACTGCCAC. Finally, FVB mice carrying the *MMTV- neu* transgene [53] (FVB/N-Tg(MMTVneu)202Mul/J) were obtained from Jackson Labs (Bar Harbor, MN). Presence of the *neu* transgene was verified using the forward primer TTTCCTGCAGCAGCCTACGC and the reverse primer CGGAACCCACATCAGGCC. The double compound transgenic mice were then crossed with the *MMTV-neu* transgenic mice to obtain triple compound transgenics, screened using the three primer sets detailed above. Females were used for mammary tumor analysis.

### Tumor analysis

Mice were bred to yield 46 triple compound transgenic females. Twenty-three of these were administered doxycycline in their feed beginning at 21 days of age as described above, while 23 received no doxycycline and acted as controls. All mice were weighed and palpated weekly for the presence of mammary tumors. Kaplan-Meier tumor-free survival analysis was performed using the PAST program (P.D. Ryan and Ø. Hammer, University of Oslo) comparing the treatment group with controls (23 mice per group for more than 25 weeks of treatment). P-value for the Kaplan-Meier curve was calculated using the Wilcoxon method. Differences were considered statistically significant at *p* < 0.05. Tumors were weighed then cut in half. One half was placed in formalin prior to histological analysis, the other half was snap frozen in liquid nitrogen and stored at −80. Significance of tumor weight differences between treated and untreated animals was determined using an unpaired Student t-test.

### Effect of *mda-7/IL-24* expression on growth of transplanted tumor cells

Lineage-depleted tumor cells were isolated from triple compound transgenic mice that had not been treated with doxycycline according to an established procedure [73, 74]. Eight six week-old FVB female mice were then injected with 10,000 cells into the No 4 mammary gland fat pads under isofluorane anaesthesia. Four of the mice were administered doxycycline in their feed as described above, and 4 mice were given feed with no doxycycline as controls. The mice were euthanized 12 weeks after transplantation and the tumors were dissected and weighed. A similar experiment was performed using the double compound transgenic mice as recipients of the tumor cells.

### Effect of *mda-7/IL-24* expression on the growth of tumors in Tg mice

Ten triple compound transgenic mice not treated with doxycycline, in which small mammary tumors (<0.25 cm) had been detected, were randomized into two groups. Five mice received doxycycline in their feed as described above and 5 received the diet with no doxycycline. After 6 weeks, the mice were euthanized and tumors collected for analysis.

### Animal care

Animal protocols were approved by the University of Toronto in accordance with the guidelines of the Canadian Council of Animal Care.

### Western blotting

Western blotting was conducted as described [44]. Polyclonal rabbit anti-rat IL-24 antibodies were obtained from GenHunter (dilution of 1:1,000); β-actin from Sigma (dilution of 1:50,000).

### Q-rtPCR amplification

RNA levels were quantified by the real-time rtPCR (q-rtPCR) in a StepOne Plus thermal cycler (Applied Biosystems) using specific primers and TaqMan probes (Genomed), as described [44]. The β-actin gene was used as control; SuperReal PreMix Plus SYBR^®^Green (TianGen, Beijing) was used for detection. The primers for PERP amplification were: sense-TCTTCCTTTAGTGGCGGTGT, anti-sense-ACGTCTGGATGTGGTTGCTA-3.

### Cloning and transduction

Transduction of *mda-7/IL-24* or vector alone into SKBR3 cells was previously described [43, 44]. Expression of *mda-7/Il-24* in these cells was confirmed by Q-rt-PCR [43, 44]. The human *perp* gene was isolated from human peripheral blood mononuclear cells (PBMC) by PCR using forward: ATCTCGAGCAGGCCACTCTCTGCTGTC and Reverse: CTCCCACATTCATTCCCAAGT primers. The cDNA was first cloned into pCR2.1 plasmid using TA cloning kit (Invitrogen) and then subcloned into XhoI and EcoRI sites of the retroviral vector MSCV2.2 [43]. MSCV-perp and vector was then transfected into SKBR3 cells, as described [43]. The expression of *prep* in the transduced cells was determined using Q-rt-PCR using forward:ATAACTGGGCCTACGGCTTT and Reverse:CTCCCACATTCATTCCCAAGT primers.

### Luciferase assay

pPERPluc1 was purchased from addgene and deposited by Dr. Tyler Jacks, as described [75]. This plasmid (2μg) was transfected into FE1.2+IL-24 and FE1.2+vector cells, using lipofectamine 2000 (Life technology, Beijing, China). After 48 h of transfection, luciferase assays were performed in triplicate as described [75, 76].
